# Instant Endocarditis Diagnosis Using Point-of-Care Ultrasound (POCUS) in a Patient Diagnosed With Pneumonia

**DOI:** 10.7759/cureus.36043

**Published:** 2023-03-12

**Authors:** Larry Istrail

**Affiliations:** 1 Hospital Medicine, Inova Fairfax Hospital, Falls Church, USA

**Keywords:** 2d echocardiography, cardiology imaging, pocus (point of care ultrasound, tricuspid valve endocarditis, point-of-care-ultrasound

## Abstract

Acute bacterial endocarditis is an acute febrile illness that spreads hematogenously and can be fatal if it is not treated in a timely fashion. A traditional physical examination has very limited sensitivity and specificity when diagnosing bacterial endocarditis. Point-of-care ultrasound (POCUS) during the physical exam can assist with the diagnosis by evaluating for valvular regurgitation or visible vegetation. In this case, a patient presented to the hospital with a cough and shortness of breath and was diagnosed with pneumonia. She did not improve with intravenous antibiotics and a POCUS exam revealed the diagnosis was in fact bacterial endocarditis and not pneumonia. This led to further imaging, which revealed an abdominal abscess. This highlights the importance of incorporating POCUS into the physical exam of any patient presenting with cardiopulmonary symptoms.

## Introduction

Acute bacterial endocarditis is a febrile illness that spreads hematogenously and can be fatal if not treated promptly [1]. Since endocarditis commonly presents with non-specific symptoms like malaise, as any other bacterial infection may, it can be very difficult to diagnose. Point-of-care ultrasound (POCUS) during the physical exam can assist with the diagnosis by evaluating for valvular regurgitation or visible vegetation. While the sensitivity of detecting valvular endocarditis with POCUS or transthoracic echocardiography is relatively low at about 60%, the specificity is over 90% [2,3]. Therefore if endocarditis is suspected, a POCUS exam can be useful to rule in endocarditis if vegetation or new severe valvular dysfunction is visualized.

## Case presentation

A 69-year-old female with a history of hypertension, recent *E. coli *bacteremia, and hiatal hernia presented to the hospital with a nonproductive cough and progressive dyspnea for two months. Three months prior to admission, she was admitted to the hospital with fevers and was found to have *E. coli* bacteremia. She completed a course of intravenous antibiotics, and her symptoms resolved completely. One week prior to admission, she visited her pulmonologist for her persistent cough, who prescribed a course of levofloxacin; however, her symptoms did not improve and she presented to the hospital.

**Figure 1:**
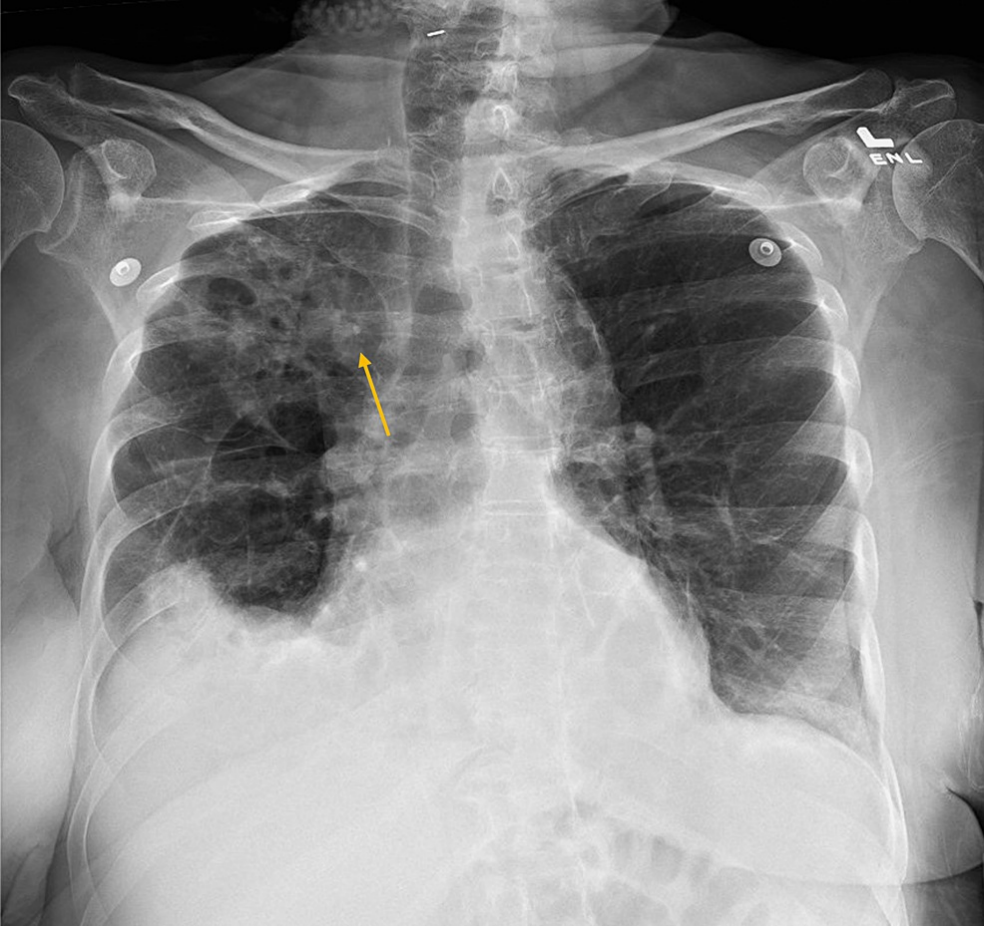
Chest X-ray (anterior to posterior view) The arrow shows right upper lobe consolidation.

On arrival at the emergency department, she was afebrile with a heart rate of 86 beats per minute and a blood pressure of 130/89 mmHg. Labs were significant for a leukocytosis of 15,000 WBCs per uL and hemoglobin of 8.7 g/dL. The basic metabolic panel was non-contributory. The procalcitonin was mildly elevated at 0.6 ng/ml. An anterior to posterior (AP) chest X-ray in the emergency department revealed ​​multifocal right-sided airspace opacities and right effusion (Figure 1).

**Figure 2:**
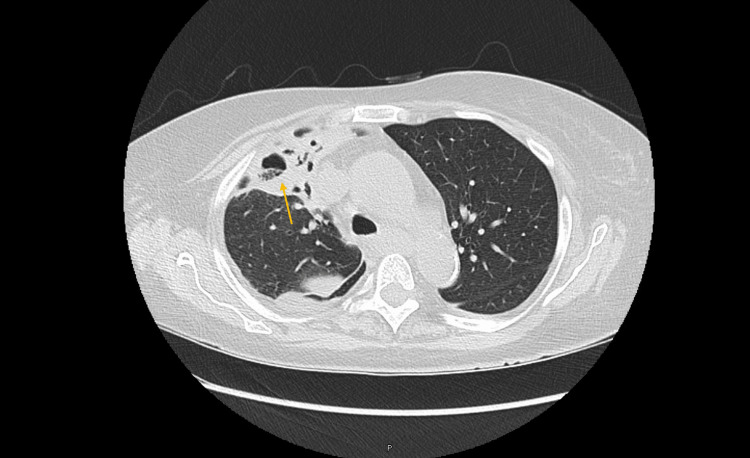
CT chest on admission The yellow arrow shows large right upper lobe consolidation.

A subsequent CT scan of her chest revealed multifocal cavitating opacities in the right upper and lower lobes, with additional smaller peribronchial nodular opacities in all five lobes. The radiologist suspected these findings were consistent with an atypical infectious process or chronic aspiration pneumonia with necrosis (Figure 2).

She was started on broad-spectrum antibiotics and admitted to the hospital for ongoing treatment. On day two of hospitalization, she was seen by the internal medicine team. She declined to wear a hospital gown and declined a cardiopulmonary point-of-care ultrasound (POCUS) examination. She was seen by pulmonology who suspected her findings were consistent with aspiration pneumonia in the setting of her chronic hiatal hernia. They recommended continued antibiotics with ampicillin-sulbactam and a gastroenterology consultation. On day two of hospitalization, she was seen by the gastroenterology team, who recommended esophagogastroduodenoscopy (EGD) once her pneumonia improved.

On day three of hospitalization, her symptoms persisted and her heart rate continued to rise despite intravenous antibiotics. An EKG revealed sinus tachycardia with a heart rate of 125 beats per minute. Her COVID-19 and influenza PCR (polymerase chain reaction) tests were negative, and her blood cultures showed no growth. On day four of her hospitalization, pulmonology recommended a transition from intravenous (IV) antibiotics to oral augmentin for a 21-day extended course in preparation for hospital discharge.

Given her persistent sinus tachycardia, she was strongly encouraged by this author to allow a POCUS exam and she reluctantly agreed. POCUS exam of her jugular vein revealed jugular venous distention (JVD) 2 cm above the clavicle with the patient’s head of the bed at 45 degrees consistent with mildly elevated right atrial pressure. POCUS lung exam revealed a small right pleural effusion, anterior focal B-lines, and a large hypoechoic subpleural consolidation consistent with her CT findings. The cardiac POCUS exam in parasternal long and parasternal short axis views revealed a hyperdynamic ejection fraction with no evidence of pericardial effusion (Videos 1-2).

**Video 1:** Parasternal long axis view showing hyperdynamic ejection fraction

**Video 2:** Endocarditis (parasternal short axis view) Parasternal short axis view showing a hyperdynamic ejection fraction. Vegetation cannot be seen in this view.

The apical 4-chamber view revealed a possible mobile density on the tricuspid valve concerning for tricuspid valve endocarditis (Video 3). A STAT comprehensive echocardiogram was ordered which confirmed a medium mobile echodensity on the tricuspid valve septal leaflet extending back and forth during the cardiac cycle consistent with vegetation. The cardiothoracic surgery team was consulted but no surgical intervention was recommended since only mild tricuspid regurgitation was present.

**Video 3:** Mobile mass seen on tricuspid valve In this apical 4-chamber view, a hyperechoic mass can be seen attached to the tricuspid valve.

With negative blood cultures, the source of her endocarditis remained unclear. On the fifth day of hospitalization, a CT scan of her abdomen and pelvis was obtained, which revealed a 4 x 2 x 4 cm left lower quadrant abscess due to complicated diverticulitis of the descending colon. Her antibiotics were narrowed to ceftriaxone and metronidazole, and on day six of her hospitalization, she underwent a CT-guided abdominal abscess drainage. Neither her initial blood cultures, repeat blood cultures, nor her abscess drainage showed any bacterial growth. Her sinus tachycardia resolved and she was discharged with four weeks of infective endocarditis therapy with metronidazole and ceftriaxone.

## Discussion

Endocarditis is a difficult-to-diagnose condition that sometimes arises in bacteremic patients. Without POCUS or transthoracic echo, the diagnosis of endocarditis must rely on physical exam findings that are neither sensitive nor specific, such as auscultation for new murmurs or inspection of the skin for splinter hemorrhages or Janeway lesions [4]. Compared to transesophageal echo (TEE), transthoracic echo (TTE) has low sensitivity for detecting endocarditis. This sensitivity is limited by body habitus, available cardiac windows, and further limited with a POCUS exam due to generally lower resolution images [5]. However, the specificity is over 90%, so when a mobile mass is detected, it is very likely due to endocarditis.

In this case, the patient presented in a very atypical fashion with shortness of breath and cough as the result of downstream manifestations of an abdominal abscess with presumed bacteremia. In the presence of negative blood cultures, the endocarditis must be treated empirically while searching for a source. The most common causes of infective endocarditis are *Staphylococcus aureus*, *Streptococcus viridans*, coagulase-negative staphylococci, *Enterococcus*, and *Streptococcus bovis* [6]. This patient’s recent *E. coli* bacteremia made *E. coli* a possible cause, though this is an extremely rare cause of infective endocarditis. In an 18-year prospective survey of 3605 bacteremia episodes, *E. coli* comprised 23.9% of them, and only two patients developed infectious endocarditis [7]. The endocarditis would have likely gone undetected in this patient with no cardiac history, and therefore POCUS was essential to discover the underlying diagnosis.

## Conclusions

As demonstrated in this case, POCUS examinations can drastically alter a patient’s clinical course. Despite CT imaging of the lungs, pulmonology consultation, and IV antibiotics, the patient was not improving. POCUS enabled instant bedside visualization of the jugular vein, heart, and lungs, providing clues of active infection and cardiac dysfunction that led to the underlying diagnosis. A POCUS exam that does not visualize a mobile density cannot rule out endocarditis, though if it is seen with POCUS, it can confirm the diagnosis. Understanding that POCUS offers high specificity and low sensitivity in the case of infective endocarditis is important to understand when using the results to guide clinical decision-making.
